# Lower Extremity Asymmetry Values Derived From Multiple Strength Testing Modes Are Associated With Perceived Functional Capabilities Among University Athletes

**DOI:** 10.1155/2024/5589056

**Published:** 2024-10-23

**Authors:** Zachary J. McClean, Nathan Boon-van Mossel, Mark McKenzie, Per Aagaard, Walter Herzog, Kati Pasanen, Victor Lun, Matthew J. Jordan

**Affiliations:** ^1^Faculty of Kinesiology, University of Calgary, Calgary, Canada; ^2^Integrative Neuromuscular Sport Performance Lab, Faculty of Kinesiology, University of Calgary, Calgary, Canada; ^3^Department of Sport Science and Clinical Biomechanics, University of Southern Denmark, Odense, Denmark; ^4^Sport Medicine Centre, University of Calgary, Calgary, Canada

**Keywords:** athlete introspective measures, athlete performance, between-limb asymmetry, biopsychosocial, injury prevention

## Abstract

**Background:** Muscle strength asymmetry and athlete introspective measures are associated with musculoskeletal (MSK) sport injury and reinjury. However, the interrelationship between mechanical and subjective measures of concentric and eccentric limb function needs further exploration. This includes investigating if an athlete's perception of their overall MSK function influences limb asymmetry across different testing modalities.

**Objectives:** To (i) explore the interrelationship between mechanical and subjective measures of lower limb function in university athletes and (ii) compare the consistency in interlimb strength asymmetries across different strength tests in groups of athletes with good, fair, and poor perceived limb function.

**Methods:** University athletes (*n* = 175; *n* = 87 females) from six sports completed four tests of muscle strength, power, and plyometric function along with an assessment of perceived limb function using the Sport Fitness Index (SFI). Participants were categorized into high (GOOD_SFI_), fair (FAIR_SFI_), and poor (POOR_SFI_) perceived overall MSK function (perceived function) groups. Strength asymmetry indexes evaluated interlimb differences in concentric and eccentric maximal strength, countermovement jump (CMJ) impulse, unilateral CMJ height, and reactive strength index in unilateral repeat hop testing. Cumulative link mixed-effects models assessed the relationship between strength asymmetries and perceived limb function.

**Results:** The POOR_SFI_ group showed increased asymmetry in concentric strength testing (*p* = 0.022), more consistent interlimb asymmetries (*p* < 0.001), and reduced overall muscle strength compared to the GOOD_SFI_ group.

**Conclusion:** Higher interlimb asymmetry in maximal concentric strength measures along with reduced muscle strength was found in the POOR_SFI_ group compared to the GOOD_SFI_ group. The POOR_SFI_ group also showed greater consistency in muscle strength asymmetry derived across different tasks.

## 1. Introduction

Previous musculoskeletal (MSK) sport injury is a risk factor for future injury [1], in part due to chronic impairments in neuromuscular function and diminished psychological readiness stemming from incomplete postinjury rehabilitation [2]. To address the impact of sport injury on athlete health and performance, preseason baseline mechanical and psychological assessments such as the Sport Fitness Index (SFI) [3, 4] have been recommended to identify athletes with compromised neuromuscular or psychophysiological function [5].

The SFI is a validated survey used to quantify the effects of previous injury on factors such as limb function that influence an athlete's perception of their overall MSK function through the evaluation of associated symptomatology related to sport injury including reduced neuromuscular function and impaired psychological readiness [4, 6–8]. The SFI is commonly implemented to screen athletes who may be at increased risk for sport injury [3, 4]. Not only can the SFI identify athletes who have inadequately recovered from previous injury, but it may also flag athletes with functional and psychological deficits associated with factors such as a poor training history that are not detected in typical preseason injury screening processes [3]. Questionnaires such as the SFI that assess psychological markers associated with sport injury through introspection align with a broader theme in athlete monitoring research to incorporate a biopsychosocial approach to injury prevention research [9]. Although recent literature has highlighted a potential association between an athlete's introspective state and biomechanical limb asymmetries [10], further exploration of the interrelationship between introspective, psychological, and biomechanical markers of asymmetry across eccentric and concentric muscle actions is needed. This is particularly relevant for university athletes who are exposed to a unique set of psychosocial stressors and are at high risk for lower body MSK sport injury. This is important because combining mechanical and introspective tests of limb function may help clinicians and practitioners to intervene with targeted training and rehabilitation tailored to the needs of a high-risk athlete.

Athletes require different types of strength to perform in their sport, so lower limb mechanical muscle strength testing is typically performed using a battery of neuromuscular assessments that evaluate high-force muscle strength, maximal muscle power, and plyometric function [5]. Muscle strength can also be evaluated in different contraction types (eccentric, isometric, and concentric muscle actions). The strength of each limb is often compared using an interlimb asymmetry index (AI) calculation [11], where increased asymmetry may reflect a performance deficit or the adoption of a high-risk movement strategy [12]. Various testing modalities have been used to assess interlimb asymmetry in athletes including tests of dynamic movement control [13, 14], maximal muscle power [10, 15], muscle strength [16, 17], and plyometric function [18, 19]. While the magnitude and directionality of interlimb strength asymmetries across different functional tests appear to be task dependent in noninjured athletes [20, 21], the interrelationship as a function of perceived limb function has not been studied. This line of inquiry is important as it promotes a more holistic understanding of biological–psychological baseline testing methodologies to identify athletes at increased risk for sport injury.

The overarching aim of this study, therefore, was to increase the scientific understanding on the interrelationship between mechanical and subjective measures of lower limb function in university athletes. We achieved this by first evaluating the relationship between perceived limb function group status and interlimb strength asymmetries across a range of neuromuscular tests designed to evaluate muscle strength, power, and plyometric capacity. Next, we compared the consistency in interlimb strength asymmetries across these different tests in groups of athletes with good, fair, and poor perceived limb function. We hypothesized that increased muscle strength asymmetry would predict poorer perceived limb function. Additionally, we hypothesized that muscle strength asymmetries would be task independent (i.e., demonstrate greater consistency) in the poor perceived function group compared to the good perceived function group.

## 2. Methods

### 2.1. Participants

University athletes (*n* = 195; females: *n* = 96 (49.2%)) from six high-risk sports (soccer, basketball, volleyball, tackle football, rugby, and field hockey) were recruited for the study using cluster sampling (Table 1). Prior to testing, all participants provided their written informed consent. The inclusion criteria for this study were that participants must (i) be preparing for competition in the upcoming university sport season; (ii) not have been diagnosed with a traumatic brain injury within the last 12 months; and (ii) be cleared to perform the neuromuscular assessments. Twenty participants who did not complete the complete multicomponent strength testing battery were excluded from the data analysis. Participants were excluded if they were unable to grip and hold the hexagonal bar during loaded Countermovement Jump (CMJ) testing, and if they declined to perform the unilateral testing on one side, preventing an AI calculation.

Therefore, 175 participants (females: *n* = 87 (49.7%)) were included for the final analysis. The protocol for this study was approved by a Research Ethics Board (REB15-1094) and conducted in accordance with the Declaration of Helsinki (without registration).

**Figure 1:**
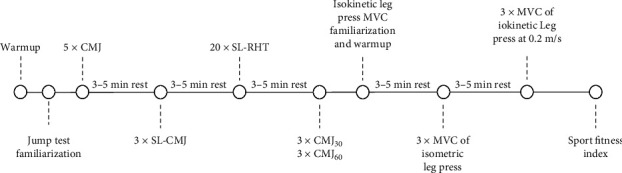
Overview of testing procedures. CMJ, countermovement jump; CMJ_30_, countermovement jump with 30% body mass; CMJ_60_, countermovement jump with 60% body mass; SL-CMJ, single-leg countermovement jump; SL-RHT, singe-leg repeat hop test; min, minutes; MVC, maximum voluntary contraction.

An overview of the testing procedures is shown in Figure 1. A qualified exercise physiologist familiarized participants with the neuromuscular assessments. Prior to baseline preseason strength testing, participants completed a standardized 10-min warmup protocol at a self-selected moderate intensity on a cycle ergometer followed by 4 × 10 s maximal cycling sprints interspersed with 50 s of rest. Standardized rest periods between strength testing protocols (3–5 min) were included in the study design to minimize any potential effect of fatigue during the protocol. Immediately following (∼5 min) strength testing, participants completed an electronic survey that included participant demographic information (i.e., sex, gender, age) and the SFI [4].

### 2.2. CMJ Testing

Participants performed five maximal bilateral CMJ repetitions with each foot placed on a force plate, their hands placed on the hips, and while descending rapidly to a self-selected depth [22]. Each jump was separated by 5 s of quiet standing while they awaited a strong verbal cue by the researcher to start the next CMJ repetition. Loaded CMJ trials were performed in the same manner while holding a hexagonal bar with an extra external load equal to 30% (CMJ_30_; *n* = 3 repetitions) and 60% (CMJ_60_; *n* = 3 repetitions) of their body mass. Finally, three maximal single-leg CMJs (SL-CMJ; *n* = 3 repetitions) were performed on each leg using the protocol described above.

CMJ testing was performed on a dual force plate system (Accupower, Advanced Measurement Technology Instruments, Boston, Massachusetts, USA). The vertical ground reaction force (Fz) from the right and left legs was measured synchronously at a sampling frequency of 1000 Hz and stored on a personal computer (Noraxon myoRESEARCH version 3.20, Scottsdale, AZ, USA). Data were exported and analyzed using a custom-built computer program (MATLAB R2022a, MathWorks, Natick, MA, USA) as described previously [15, 23, 24]. Discrete CMJ movement phases were defined according to the velocity of the body center of mass (BCM), obtained by time integration of the instantaneous acceleration signal calculated from the total (bilateral) Fz [15, 22] and assuming that the vertical velocity for all tests was zero at the start of the jumping tests. The participant's body mass was calculated as the total (bilateral) Fz during a 2-s quiet standing period [15]. The eccentric deceleration phase was defined as the time interval from maximal negative BCM velocity to zero velocity [15]. The concentric phase was defined from the lowest BCM position to the instant of the vertical takeoff (toe-off) [15]. Time integration of Fz was used to calculate the impulses for the right and left limbs across the discrete movement phases [15]. Jump height was determined from vertical BCM velocity at the instant of ground toe-off (jump height = takeoff velocity^2^/(2 g)). The peak external mechanical power was calculated as the maximum value obtained from the instantaneous product of Fz and BCM velocity.

### 2.3. Single-Leg Repeat Hop Test (SL-RHT)

Participants performed twenty repetitions of the SL-RHT on each leg on the same force plate system. Participants were instructed to maximize the jump height and minimize ground contact time while keeping their hands firmly placed on the hips. The force–time signal was filtered using a bidirectional low-pass fourth-order Butterworth filter (frequency = 50 Hz). The midpoint of the flight phase of each hop was identified and assumed to represent maximal jump height with a BCM velocity of 0 m/s. The drop height (i.e., when BCM velocity = 0 m/s) of the previous hop was then used to determine the landing velocity of the subsequent hop so that discrete hopping movement phases could be calculated using the BCM velocity. The five single-leg hops with the highest reactive strength index (RSI = hop height [cm]/ground contact time [s]) values were identified for statistical analysis.

### 2.4. Single-Leg Isokinetic Leg Extension Testing

Participants performed three single-leg isokinetic multijoint leg press movements in a robotic servomotor leg press device (Fv Seger, Treadmetrix, Park City, Utah; sampling frequency = 200 Hz) to quantify multijoint maximal concentric and eccentric leg extensor strength. Here, leg extensor strength is defined as extension of the hip and knee, but not the ankle due to the typical constraints associated with a leg press. The participants laid on a recumbent chair, placed along a 2-dimensional track within the apparatus, with their feet placed on the leg press platform in a standardized position. Isokinetic testing was performed between 100 and 10 degrees of knee flexion at 0.2 m/s for both concentric (leg extension) and eccentric (leg flexion) muscle actions interspersed by a 0.5-s pause between the concentric and eccentric movement phases. Prior to testing, a task-specific warmup protocol was completed in which participants performed five concentric–eccentric isokinetic repetitions at progressively increasing submaximal intensities, along with three maximal unilateral isometric muscle contractions at a 60-degree knee flexion angle. Following a 3- to 5-min rest period, participants completed three maximal voluntary contractions (MVCs) on the isokinetic leg press with strong, standardized verbal encouragement.

The servomotor torque was converted into force (N) (representing the force exerted by the participant's foot on the leg extension machine) and low-pass filter using a fourth-order recursive Butterworth filter (cutoff frequency = 6 Hz) according to the manufacturer's recommendations. The acceleration phase of the isokinetic MVC was removed via the manufacturer's software to obtain a stable isokinetic period across the leg press range of motion. Each force–time curve was visually inspected by the researchers to exclude erroneous trials. Eccentric and concentric strength was defined as the maximal instantaneous force produced across all repetitions of the associated muscle contraction. Furthermore, the force–time curve was integrated across this time interval to obtain the contractile impulse for the eccentric and concentric MVCs of each repetition. The highest concentric and eccentric contractile impulses were obtained and used to calculate the interlimb AI (see below).

### 2.5. Interlimb AI Calculations

The interlimb AI was calculated using the following formula [15, 25]:

(1) $$\begin{array}{c} \text{AI}\left(\%\right)=\frac{\text{right limb}-\text{left limb}}{\text{maximum of right and left}}\times 100\%, \end{array}$$

where a positive (right-leg dominance) or negative (left-leg dominance) value indicated the interlimb AI directionality. The phase-specific kinetic impulse asymmetry was assessed for the bilateral CMJ protocols, which was calculated from the eccentric deceleration impulse (CMJ Bodyweight: CMJ Ecc AI; CMJ 30% extra load: CMJ_30_ Ecc AI; CMJ 60% extra load: CMJ_60_ Ecc AI) and the concentric impulse (CMJ Bodyweight: CMJ Con AI; CMJ 30% extra load: CMJ_30_ Con AI; CMJ 60% extra load: CMJ_60_ Con AI) as previously defined [15, 26]. Vertical jump height was used to calculate the SL-CMJ Height AI. The RSI was used to quantify between-limb asymmetry for the SL-RHT (RHT RSI AI). All jump asymmetries were calculated as an average value across all repetitions. For the multijoint isokinetic leg press test, the AIs were calculated from the repetition with the highest eccentric contractile impulse (MVC Ecc AI) and the highest concentric contractile impulse (MVC Con AI), respectively.

### 2.6. SFI Calculations

Each question from the original SFI was numerically coded on a 0–5 scale, these values were summed and multiplied by two to compute a score on a scale from 0 to 100 for each participant (higher scores reflecting better overall MSK function, which for the purpose of this study will be referred to as perceived function) [4]. Based on previously established cut points for predicting high risk for injury [4] and reduced functional status [3], an SFI cut point of ≥ 86 points was used to differentiate between participants with better and worse perceived function along with a cut point of < 60 points to determine participants with a very low SFI score [4]. Eighty-four (48.0%) participants were assigned to the GOOD_SFI_ perceived function group (i.e., SFI score ≥ 86 or had no self-reported lower body injury in the past 12 months), 39 participants (22.2%) were assigned to the FAIR_SFI_ perceived function group (i.e., SFI score between 60 and 85, and a self-reported lower body injury in the past 12 months), and 52 (29.7%) participants were characterized by POOR_SFI_ perceived function group (i.e., SFI score < 60, and a self-reported injury in the past 12 months).

### 2.7. Statistical Analysis

Descriptive statistics are presented as means ± standard deviation. All statistical analyses were conducted using R: A Language and Environment for Statistical Computing (v. 4.3.1). For all applicable statistics, an alpha level of 0.05 was used to determine statistical significance. A cumulative link mixed-effects model was used to assess the effects of the interlimb strength asymmetries (quantified as the AIs) on perceived limb function group status determined on a three-level ordinal scale (levels: GOOD_SFI_, FAIR_SFI_, POOR_SFI_) using the “ordinal” package (Version 2022.11–16) for R. Using backward elimination, cumulative link mixed-effects submodels were compared based on the Akaike Information Criterion (AIC). Multicollinearity for the fixed effects was evaluated using variance inflation factor from the “performance” package for R; no variance inflation factor was greater than 10, and therefore, no fixed effects were removed from the model based on these criteria. For the final model, the odds ratios and associated 95% confidence intervals (CI) were determined to assess the effects of interest. In addition, a linear mixed-effect model was used to assess the relationship between body mass normalized concentric strength, eccentric strength, and CMJ peak power output (Con MVC, Ecc MVC, and CMJ peak power) and perceived limb function group status.

To evaluate the consistency in the relationship between the strength AIs across tests for each perceived function group (GOOD_SFI_, FAIR_SFI_, POOR_SFI_), Pearson correlation coefficients (*r*) and associated *p* values were calculated for each pair of tests using the “Hmisc” package (version 5.1–1) for R. Here, *r* ≤ 0.09 was considered a *negligible* correlation, 0.1 < *r* ≤ 0.39 was considered a *weak* correlation, 0.4 < *r* ≤ 0.69 was considered a *moderate* correlation, 0.7 < *r* ≤ 0.89 was considered a *strong* correlation, and *r* ≥ 0.9 was considered a *very strong* correlation [27]. A chi-squared test with post hoc pairwise comparisons using standardized residuals was conducted to compare the proportion of tests showing weak, moderate, strong, and very strong correlations across the strength AIs for each test modality in each of the groups.

## 3. Results

**Figure 2:**
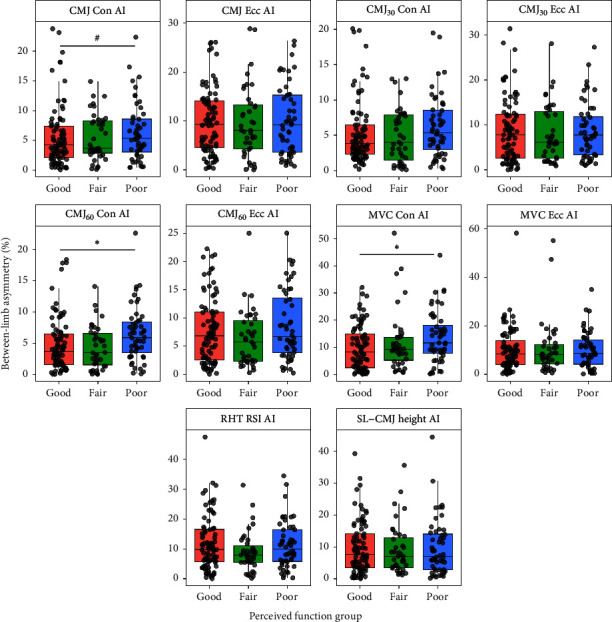
Strength asymmetry index (AI) values (%) across strength testing methods ⁣^∗^*p* < 0.05; ^#^*p* = 0.110. Con, concentric; CMJ, countermovement jump; CMJ_30_, countermovement jump with 30% body mass; CMJ_60_, countermovement jump with 60% body mass; Ecc, eccentric; MVC, maximum voluntary contraction; RHT RSI, repeat hop test reactive strength index; SL-CMJ, single-leg countermovement jump.

The magnitude of the CMJ concentric impulse asymmetry (bodyweight condition), the CMJ concentric impulse asymmetry with an extra load of 60% of body mass (CMJ_60_), and the concentric MVC contractile impulse asymmetry in the isokinetic leg press test, respectively, predicted the perceived limb function group status (*χ*^2^(3) = 9.73, *p* = 0.021, *R*^2^ = 0.027). There was a significant effect of the CMJ_60_ concentric impulse asymmetry (*β* = 0.122, SE = 0.056, *p* = 0.028) and for concentric MVC contractile impulse asymmetry (*β* = 0.035, SE = 0.016, *p* = 0.028) on perceived limb function status, whereas no significant effects were found for the CMJ concentric impulse asymmetry with bodyweight (*β* = −0.080, SE = 0.050, *p* = 0.110) (Figure 2). Thus, for every absolute percent increase in the CMJ_60_ concentric impulse asymmetry and concentric MVC contractile impulse asymmetry, the odds of belonging to a lower perceived function group increased by a factor of 1.130 (95% CI: 1.014–1.262) and a factor of 1.035 (95% CI: 1.004–1.069), respectively. Figure 2 describes the distribution of athlete AIs across all strength tests.

**Figure 3:**
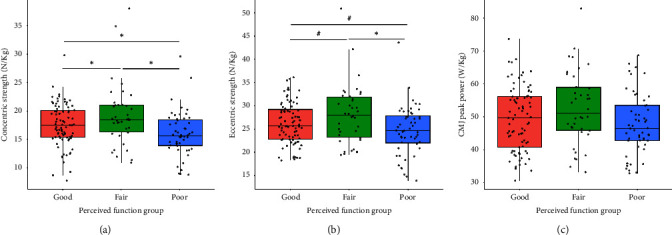
Muscle strength and power performance values across perceived limb function group. ⁣^∗^*p* < 0.05; ^#^0.110 > *p* > 0.05. CMJ, countermovement jump.

Concentric strength differed between groups (*F*_2170_ = 6.465, *p* = 0.002, *R*^2^ = 0.060) and was highest in the FAIR_SFI_ (19.2 ± 5.5 N/kg) group compared to the GOOD_SFI_ (17.5 ± 3.7 N/kg, *p* = 0.045) and POOR_SFI_ (15.9 ± 3.9 N/kg, *p* < 0.001) groups (Figure 3). Concentric strength was also higher in the GOOD_SFI_ versus the POOR_SFI_ group (*p* = 0.039) (Figure 3(a)). With reference to eccentric strength, a significant relationship was observed (*F*_2170_ = 4.612, *p* = 0.011, *R*^2^ = 0.011) where the FAIR_SFI_ group (28.1 ± 6.6 N/kg) was stronger than the POOR_SFI_ group (24.7 ± 5.3 N/kg, *p* = 0.002). No group differences in CMJ peak power were found (*F*_2170_ = 2.762, *p* = 0.066, *R*^2^ = 0.020) (Figure 3(c)).

**Figure 4:**
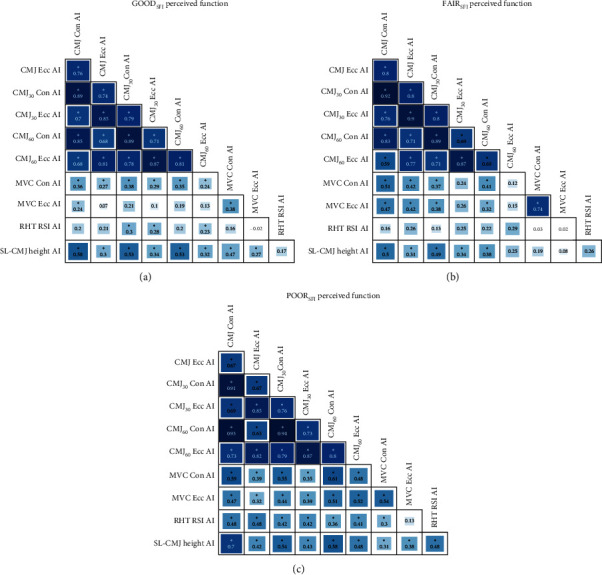
Correlation matrix depicting the relationship between strength asymmetry index values for the GOOD perceived function group (a), the FAIR perceived function group (b), and the POOR perceived function group (c). ⁣^∗^Significant correlations based on an alpha level of 0.05; CMJ Ecc AI: body weight bilateral countermovement jump (CMJ) eccentric kinetic impulse asymmetry index (AI); CMJ Con AI: body weight bilateral CMJ concentric kinetic impulse AI; CMJ_30_ Ecc AI: bilateral CMJ with 30% body mass (BM) extra load eccentric kinetic impulse AI; CMJ_30_ Con AI: bilateral CMJ with 30% BM extra load concentric kinetic impulse AI; CMJ_60_ Ecc AI: bilateral CMJ with 60% BM extra load eccentric kinetic impulse AI; CMJ_60_ Con AI: bilateral CMJ with 60% BM extra load concentric kinetic impulse AI; MVC Ecc AI: maximal isokinetic leg press eccentric impulse AI; MVC Con AI: maximal isokinetic leg press concentric impulse AI; RHT RSI AI: repeat hop test reactive strength index AI; SL-CMJ Height AI: single-leg CMJ height AI.

Figure 4 shows a multivariable correlation plot of the consistency in strength asymmetries, stratified by the GOOD_SFI_, FAIR_SFI_, and POOR_SFI_ groups. The proportion of tests with *weak*, *moderate*, *strong,* and *very strong* correlations differed between perceived function groups (*χ*^2^(6) = 26.188, *p* < 0.001) (Figure 4). Consistent with the hypothesis, a larger proportion of asymmetry tests showed a *moderate* and *very strong* correlation in the POOR_SFI_ group (53% *moderate*, 7% *very strong* correlations; Figure 4(c)) compared to the GOOD_SFI_ (13% *moderate*, 0% *very strong* correlations; Figure 4(a)) and FAIR_SFI_ (22% *moderate*, 4% *very strong* correlations; Figure 4(b)) (Table 2).

## 4. Discussion

This study examined the relationship between mechanical function and the athlete's overall perceived MSK function (perceived function) in university athletes from six high-risk sports for lower limb injury. This adds new insights into the interrelationship between mechanical and psychological neuromuscular markers related to injury risk. The primary finding of this study supported the study hypothesis and demonstrated that increased asymmetry in the concentric movement phase of the CMJ, CMJ_60_, and isokinetic MVC strength tests predicted perceived limb function group status, whereas the eccentric and unilateral AIs were not associated with perceived function. In addition to between-limb asymmetry, we offer evidence that perceived function group status is associated with concentric and eccentric strength capacities which may provide important additional context to the evaluation of interlimb strength asymmetries. The secondary finding of this study was that muscle strength asymmetries showed less task dependency and were more highly correlated in the poor perceived function group compared to the good and fair perceived function groups.

The evaluation of mechanical strength asymmetries across a range of tests has been highlighted for its utility in informing injury prevention [28, 29], determining return to sport readiness following MSK injuries like anterior cruciate ligament (ACL) rupture [25], and evaluating athletic performance [30]. Recent literature has recommended preseason baseline testing as a foundational component of athlete monitoring [31]. As part of these multifaceted assessments, interlimb strength asymmetries are often used to discriminate between athletes with a higher and lower risk for injury. However, determining which muscle strength asymmetries are the most informative for a university athlete population as it pertains to past injury history and poor perceived limb function is unclear [12]. These challenges may in part be due to wide variations in the tools and protocols used for limb asymmetry testing [12] including the specific AI calculations employed here [20], along with a lack of control for the overall MSK functional status, which can be influenced by factors such as previous injury, rehabilitation success, and training history and was addressed in our study by examining perceived function with a validated questionnaire (SFI) previously used in a college athlete population.

The present study demonstrated that increased concentric phase asymmetry, but not eccentric phase asymmetry, during high-force strength movements (CMJ_60_ and isokinetic MVC leg press testing), in addition to maximal muscle strength capacity predicted worse perceived function in university athletes. Examining mechanical and psychological neuromuscular markers of athlete function aligns with recent recommendations to use more comprehensive and holistic athlete testing protocols [25]. The SFI has been shown to be prognostic of future MSK injury in various athlete populations [3, 4], suggesting its potential utility when used in conjunction with muscle strength testing, which has also been shown to be protective against MSK injury [32]. Furthermore, in line with previous literature that has reported an association between introspective measures and biomechanical asymmetries [10], we note that perceived MSK function measured from the SFI is linked to mechanical measures of limb function including muscle strength and muscle strength asymmetries. This finding can provide important insight into psychological factors that may influence injury risk and help practitioners provide targeted and data-informed training interventions that address both movement quality and athlete psychology. However, more research is needed to identify risk patterns that consider psychological and mechanical limb function factors in relationship with sport injuries.

The multifactorial assessment of perceived and objective MSK function implemented in this study may provide advantages over traditional injury history questionnaires, which have failed to show consistent results between strength asymmetries and injury prevalence [12, 33]. The discrepancy between the present study that revealed a relationship between increased concentric strength asymmetries and poor perceived function, and previous literature may be explained by differences in testing protocols. While previous testing of muscle strength is typically performed using open-chain, single-joint movements [34], this study incorporated closed kinetic chain, dynamic, and multijoint strength testing. Given that sport injuries including ACL ruptures typically involve closed-kinetic-chain multijoint injury mechanisms, the present testing methods may be more relevant in injury prevention research than single-joint, open-kinetic chain testing.

The present study also showed that the relationship between muscle strength asymmetry and perceived function depended on the muscle contraction type (i.e., eccentric vs. concentric muscle action). Although no effects were observed for the eccentric asymmetries, high-force concentric impulse asymmetry during bilateral CMJ testing with 60% of body mass (*β* = 0.122, SE = 0.056, *p* = 0.028) and isokinetic leg press strength (*β* = 0.035, SE = 0.016, *p* = 0.028) were associated with perceived function, while a nonsignificant relationship found for the bodyweight CMJ condition (*β* = −0.080, SE = 0.050, *p* = 0.110). This highlights the importance of muscle contraction type and external load in characterizing athletes at higher and lower injury risk, as well as the potential for force–velocity profiling, a method commonly used in sport performance testing [35], to be utilized as part of a multidimensional approach to athlete injury screening. In addition, understanding limb asymmetries within the context of muscle strength capacities has been recommended with the aim of characterizing athletes who may have achieved limb symmetry at the cost of limb strength. Our results demonstrate that differences in concentric asymmetries been GOOD_SFI_ and POOR_SFI_ groups (Figure 2) are matched by reduced concentric and eccentric maximal strength capacity in the POOR_SFI_ group compared to the GOOD_SFI_ and FAIR_SFI_ groups (Figure 3). This has implications for identifying athletes at increased risk of MSK injury risk, developing injury risk reduction training programs, and informing rehabilitation status in injured athletes.

Contrary to previous research that has shown muscle strength asymmetries are task dependent [20, 34], we found differences based on perceived function group. Notably, athletes in the poor perceived function group showed greater consistency in their muscle strength asymmetries between tests compared to the athletes with good perceived limb function. The lack of consistency across unilateral and bilateral tasks in the good perceived function group may in part be due to variations in motor control arising from altered task constraints associated with single-leg movements [34, 36]. Additionally, whereas bilateral CMJs represent a common movement pattern in sport and training (i.e., volleyball, basketball, box jumps), unilateral CMJ movements from a static starting position are less common in sports and tend to arise as a separate component of specific dynamic movements (e.g., a layup in basketball). In this regard, it is plausible that the divergent movement competency between unilateral and bilateral tasks may modulate the magnitude and direction of the AI. For these reasons, Impellizzeri and colleagues [36] noted that asymmetries in bilateral CMJ testing had higher reliability than in unilateral CMJ testing, suggesting a utility for monitoring healthy and injured athletes alike. Furthermore, previous literature in healthy participants suggests that individuals tend to have a “skill-dominant limb” and a “force-dominant limb” and that skill and task limb dominance may be task dependent [37], which may have influenced the task-dependent nature of strength asymmetries in this cohort of university athletes with good perceived limb function. However, in individuals with poor perceived function, it is plausible that the limb with higher function adopts both the “skill” and “force” roles, contributing to reduced neuromuscular function, greater interlimb asymmetry, and elevated MSK injury risk [12]. More research is warranted to explore the relationships between the consistency of strength asymmetries across athletes with differing levels of perceived muscle function to determine whether a common causal neuromuscular mechanism explains the present findings.

## 5. Perspectives

MSK injuries pose a major challenge for university sport programs and the combined use of introspective and neuromuscular measures may enhance athlete baseline testing, which is an important component of the training process. This is especially crucial since low perceived function evaluated through athlete introspection along with increased interlimb biomechanical and strength asymmetries have been implicated in MSK injury risk. Our results demonstrate the interrelation of low perceived limb function and increased muscle strength asymmetries in university athletes. Lower limb mechanical strength asymmetry was higher and concentric and eccentric lower limb strength were lower in university athletes with lower perceived limb function. Not only do these results provide new insights on how athlete baseline testing may be improved by using introspective and biomechanical testing, but also it provides important information for practitioners and clinicians who seek to establish baseline neuromuscular function benchmarks for informing return to play readiness after injury.

## 6. Limitations

The study was limited by the observational design and the fact 20 participants were excluded from the final analysis as they did not complete the full test protocol. Whether or not these participants may have presented with larger interlimb asymmetries is impossible to ascertain, and this may have impacted the results. Additionally, while analyzing these data on a group level at a baseline timepoint provides valuable insight for practitioners conducting widespread preseason athlete performance testing, it is possible that the relationship between perceived limb function and mechanical limb function is individualized, and group-based analyses do not reflect individual responses. Future research should consider the combined use of athlete introspection to quantify perceived limb function and muscle strength testing to enhance injury risk profiling in university athletes.

## 7. Conclusion

The results of this study provide evidence of the interdependency of psychological and mechanical markers of neuromuscular function in university athletes. Reduced muscle strength and jump performance along with increased between-limb asymmetries during heavy concentric actions were found in the low perceived function group compared to the high perceived function group. This highlights that the external load and contraction type are important considerations when evaluating the interrelationship between mechanical and psychological markers of limb function. These findings, along with the fact that the consistency of asymmetries across different tests was associated with perceived limb function status, may have important implications for identifying athletes at elevated risk of MSK injury.

## Figures and Tables

**Table 1:** Participant information and normative performance values: sex (male [M], female [F]), body mass, bilateral countermovement jump (CMJ) peak power normalized to body mass (W/kg), concentric and eccentric isokinetic leg press (LP) strength (peak force normalized to body mass [N/kg]), unilateral (SL) CMJ height (cm), unilateral repeat hopping reactive strength index (RSI).

Sport	Sex (M/F)	Age (years)	Body mass (kg)	CMJ peak external mechanical power output (W/kg)	Isokinetic concentric LP strength (N/kg)	Isokinetic eccentric LP strength (N/kg)	SL-CMJ height (cm)	SL-repeat hop RSI (Au)
Basketball	F (*n* = 10)	20.7 ± 1.2	73.2 ± 6.9	46.2 ± 6.2	12.5 ± 2.9	20.7 ± 4.3	13.9 ± 2.3	0.27 ± 0.05
	M (*n* = 10)	20.9 ± 2.2	89.0 ± 13.2	55.9 ± 6.3	12.0 ± 3.0	19.6 ± 3.6	18.7 ± 3.4	0.27 ± 0.06

Soccer	F (*n* = 23)	20.2 ± 1.4	64.0 ± 7.4	40.9 ± 3.9	11.2 ± 2.1	19.7 ± 3.5	12.0 ± 1.8	0.25 ± 0.06
	M (*n* = 14)	20.8 ± 1.5	73.5 ± 8.2	54.3 ± 6.8	17.1 ± 2.4	22.2 ± 4.6	19.9 ± 3.5	0.33 ± 0.07

Volleyball	F (*n* = 13)	19.9 ± 1.2	71.8 ± 6.8	50.0 ± 4.8	8.7 ± 1.5	14.0 ± 2.2	15.7 ± 2.7	0.29 ± 0.05
	M (*n* = 14)	19.8 ± 5.7	81.6 ± 8.6	58.2 ± 4.9	9.6 ± 1.9	14.6 ± 2.5	19.6 ± 2.4	0.28 ± 0.08

Tackle football	M (*n* = 61)	20.5 ± 1.6	99.7 ± 25.0	55.8 ± 9.9	14.7 ± 3.8	20.5 ± 5.2	17.4 ± 5.7	0.26 ± 1.06

Rugby	F (*n* = 29)	19.8 ± 2.0	70.2 ± 12.5	41.1 ± 6.8	14.1 ± 3.0	22.1 ± 3.7	10.4 ± 2.8	0.21 ± 0.08

Field hockey	F (*n* = 21)	20.2 ± 2.3	63.1 ± 9.8	39.3 ± 4.3	12.6 ± 2.9	20.3 ± 3.4	10.3 ± 2.1	0.24 ± 0.05

**Table 2:** The proportion (total number; *n*) and standardized residuals (Std Res) for good, fair, and poor Sport Fitness Index (SFI) derived perceived function groups (GOOD_SFI,_ FAIR_SFI_, and POOR_SFI_, respectively) across between-test correlation strengths (weak correlation: 0.1 < *r* ≤ 0.39; moderate correlation: 0.4 < *r* ≤ 0.69; strong correlation: 0.7 < *r* ≤ 0.89; very strong correlation: *r* ≥ 0.9 [27]).

Perceived function group	Weak correlation (*n* (Std Res))	Moderate correlation (*n* (Std Res))	Strong correlation (*n* (Std Res))	Very strong correlation (*n* (Std Res))
GOOD_SFI_	26 (2.33)	6 (−2.66)	13 (0.82)	0 (−1.66)
FAIR_SFI_	22 (1.53)	7 (−1.84)	10 (−0.04)	2 (0.41)
POOR_SFI_	9 (−3.84)	23 (4.48)	9 (−0.79)	3 (1.26)

## Data Availability

The data that support the findings of this study are available from the corresponding author upon reasonable request.
